# Ethics teaching in medical school: the perception of medical students

**DOI:** 10.1007/s00508-022-02127-7

**Published:** 2022-12-22

**Authors:** Lorenz Faihs, Carla Neumann-Opitz, Franz Kainberger, Christiane Druml

**Affiliations:** 1 Medical University of Vienna, Vienna, Austria; 2 Ludwig Boltzmann Institute for Experimental and Clinical Traumatology, Vienna, Austria; 3 Division of Neuro- and Musculoskeletal Radiology, Department of Biomedical Imaging and Image-Guided Therapy, Medical University of Vienna, Vienna, Austria; 4 Unesco Chair on Bioethics at the Medical University of Vienna, Vienna, Austria; 5 Department of Ethics, Collections, and History of Medicine (Josephinum), Vienna, Austria

**Keywords:** Medical ethics, Healthcare ethics, Ethics education, Ethics curriculum, COVID-19

## Abstract

**Background:**

In times of a pandemic, morals and ethics take center stage. Due to the challenges of the pandemic and ongoing discussions about the end of life, student teaching demands might have changed. This study aimed to evaluate teaching ethics, law, and decision-making skills in medical education via a survey to customize the curriculum to the students’ needs. Furthermore, gender differences were examined to determine gender equality in medical education.

**Methods:**

The medical students at the Medical University of Vienna were requested to complete an anonymous online survey, providing feedback on the teaching of ethics, law, and decision-making skills.

**Results:**

Our study showed the students’ strong demand for more teaching of ethics, law, and decision-making skills. Moreover, we found that students were afraid to encounter ethical and moral dilemmas. Gender differences could be found, with female students assessing their knowledge and the teaching as being more insufficient, resulting in greater fear of encountering ethical and moral dilemmas.

**Conclusion:**

The fear of encountering ethical and moral dilemmas might be linked to medical students’ self-perceived insufficient legal knowledge. The education should guarantee gender equality in medical training and be customized to the students to provide the future doctors with the ethical and legal expertise to preserve the patient’s rights and protect their mental health.

## Introduction

Medical knowledge and skills are undoubtedly the basis of medical education; however, with every medical decision, various aspects must be considered. Among these are ethics, morality, and legal factors, which also play a significant role in the everyday working life of a doctor. At the beginning of the Coronavirus disease 2019 (COVID-19) pandemic, with scarce resources and limited knowledge about the disease, doctors faced difficult decisions that may often contradict their personal ethical and moral principles. These personal ethical or moral code violations might lead to adverse psychological effects, such as a moral injury [1]. The symptoms include negative thoughts about oneself, such as shame, guilt, or disgust, which can lead to further mental health issues [2].

Doctors must train their intellectual and emotional capacity to develop an ethical preparedness to make decisions in times of crisis [3]. Medical schools should provide future doctors with the tools to face these difficult situations by integrating the education of medical ethics and the legal background vertically and horizontally in their curriculum [4].

The medical ethics curricula, their structural preconditions and the respective academic course hours vary substantially between medical schools [5, 6]. UNESCO created a bioethics core curriculum, which covers the core contents of the bioethical principles of the Universal Declaration on Bioethics and Human Rights [7]. Furthermore, the World Medical Association Ethics Unit published a Medical Ethics Manual to provide a “universally used curriculum for the teaching of medical ethics” [8].

At the Medical University of Vienna, bioethics is taught primarily in the public health module in the fourth year, which consists of 12 academic hours. The teaching has an interdisciplinary approach based on four points of view: ethics in the healthcare system, ethical issues in the doctor-patient relation, ethical aspects at the end of life and in palliative care, ethnomedicine and ethical aspects of the interculturality [9].

The way knowledge is tested at the Medical University of Vienna mainly consists of a summative, integrated examination (SIP), a written multiple choice test at the end of each academic year. Haidinger et al. revealed that female students showed less confidence in success before taking this examination [10]. Furthermore, past studies showed that female students perceive their skills and knowledge as inferior, although they end up being equal to their male colleagues [11, 12]. Through our study, we aimed to evaluate if a gender difference can also be found regarding the assessment and the opinions about bioethics.

Past studies have evaluated ethical competence and the students’ awareness of and attitudes towards medical ethics [13–18]; however, due to the ongoing changes in moral concepts and the current pandemic with its concomitant ethical and moral dilemmas, the students’ perception of the teaching of ethics and morals might also have evolved. We sought to assess the student’s evaluation of ethics teaching in medical education with a particular focus on the ethical and moral aspects of a pandemic and end of life issues as well as the corresponding legal background. Furthermore, we wanted to survey the students’ awareness of ethical topics, their wishes, and preferences for a better education in bioethics and to evaluate if there are any differences in opinion between genders. The findings of this study can contribute to understanding the students’ needs, wishes, and fears regarding their future working life and improve ethics teaching in medical education by implementing the results in the medical curricula.

## Methods

Medical students at the Medical University of Vienna were asked to give feedback on the ethics curriculum in an online survey. The anonymous questionnaire was embedded in the Moodle course of the lecture line interdisciplinary case conferences in which all students (*n* = 662) of the ninth semester were enrolled. The prescribed course covers ethical and moral aspects of medicine among other topics and therefore provides a good opportunity to ask for feedback regarding the survey’s topics. This cohort of students finished ethical teaching in medical school after an emphasis on bioethics in the fourth year. They will soon be confronted with moral and ethical dilemmas with the start of their clinical training in their elective work in the sixth year. We decided to conduct the survey through an online questionnaire in German to reach as many students as possible. Completing the online questionnaire was voluntary and anonymous; there were no consequences if the students did not participate in the survey. We refused to ask for age and other demographic data besides gender to preserve the anonymity of the participants.

After consultation with the intrauniversity data protection and ethics committee of the Medical University of Vienna, no formal approval was needed for this voluntary and totally anonymous online survey.

### Questionnaire

The questionnaire was developed with the input of all authors and adapted to focus on the quality of ethics teaching at the Medical University of Vienna. It included 17 statements with a 6-point Likert scale ranging from 1 = strongly agree to 6 = strongly disagree, 2 multiple choice questions (including the question of gender), and 1 open question (I would like an increased emphasis on ethical and moral aspects during medical school in the following topics). We grouped the questions and statements in four subchapters: firstly, the assessment of the education of ethics, morals, and the legal aspects, secondly, pandemics and the current COVID-19 situation, thirdly, end of life and lastly, the preferences for medical ethics curriculum.

### Data analysis

Statistical Analysis was performed with IBM SPSS Statistics 27 software (IBM Corp., Armonk, NY, USA). The responses to the open question were grouped into 20 categories to allow statistical analysis. As the data were non-normally distributed, the median and interquartile range (IQR) were given. For the comparison of responses by gender, a Mann-Whitney U‑test was performed; for the comparison of the answers of two questions, a Wilcoxon test was used. Bivariate correlations were analyzed with the Spearman rank correlation. A *p*-value of < 0.05 was considered statistically significant. No multiple comparison adjustments were performed; *p*-values should be interpreted as exploratory only.

## Results

With 662 actively enrolled students in the Moodle course interdisciplinary case conferences in January of 2021, 283 questionnaires were completed, resulting in a response rate of 42.8%. The majority of respondents identified themselves as female (59.1%, *n* = 133), 39.6% (*n* = 89) as male, and 1.3% (*n* = 3) as non-binary (*n* = 58 missing data).

Correlations are only mentioned for significantly correlating responses.

### Assessment of ethics teaching

Table 1 provides an overview of the assessment of ethics teaching by the students. It shows the frequency of the answers the students provided. The most frequently marked answer was highlighted in italic text for each question. Firstly, students tended to think that ethics and morals are sufficiently covered in medical school (IQR = 2–4, median 3 = slightly agree). Nevertheless, the students did not think physicians and professors took enough time to discuss ethical and moral issues with students (IQR = 3–5, median 4 = slightly disagree).

**Table 1:** Summary statistics of the assessment of ethics teaching

	1 Strongly agree	2 Mostly agree	3 Slightly agree	4 Slightly disagree	5 Mostly disagree	6 Strongly disagree	Missing	Overall score/mean	Median
I think that ethics and morals are sufficiently covered in medical school	8.5% (24)	22.3% (63)	22.6% (64)	*26.9%* *(76)*	15.2% (43)	4.2% (12)	0.4% (1)	3.31	3
I think that legal aspects about ethical and moral issues are sufficiently covered in medical school	7.1% (20)	11.3% (32)	18.7% (53)	*29.7%* *(84)*	23.3% (66)	8.8% (25)	1.1% (3)	3.78	4
I feel sufficiently prepared to make a decision in the event of an ethical or moral dilemma	4.9% (14)	20.1% (57)	26.1% (74)	*26.5%* *(75)*	14.5% (41)	7.1% (20)	0.7% (2)	3.47	3
I think that physicians and professors take enough time to discuss ethical and moral issues with students	7.8% (22)	14.5% (41)	22.6% (64)	*24.4%* *(69)*	21.6% (61)	8.5% (24)	0.7% (2)	3.63	4
I am afraid to encounter ethical and moral dilemmas in my future professional life	14.1% (40)	*31.8%* *(90)*	20.1% (57)	19.8% (56)	9.5% (27)	3.9% (11)	0.7% (2)	2.90	3
I think that physicians are often confronted with ethical and moral dilemmas in their professional life	*57.2%* *(162)*	33.2% (94)	7.4% (21)	0.7% (2)	1.1% (3)	0% (0)	0.4% (1)	1.55	1
I have sufficient legal knowledge to handle ethical and moral dilemmas	2.8% (8)	8.5% (24)	19.4% (55)	*27.9%* *(79)*	26.9% (76)	13.4% (38)	1.1% (3)	4.09	4
I think that decision-making skills are sufficiently taught in medical school	4.6% (13)	18.0% (51)	25.4% (72)	*29.3%* *(83)*	15.2% (43)	6.7% (19)	0.7% (2)	3.53	4

In contrast to the assessment of ethics and morals teaching, the legal aspects of ethical and moral issues were regarded as not sufficiently covered in medical school (IQR = 3–5, median 4 = slightly disagree). Furthermore, the prospective doctors mostly did not think they had sufficient legal knowledge to handle ethical and moral dilemmas (IQR = 3–5, median 4 = slightly disagree). The difference between the assessment of ethics and morals teaching and the teaching of legal aspects was statistically significant (*p* < 0.001). While most participants felt sufficiently prepared to make a decision in the event of an ethical or moral dilemma (IQR = 2–4, median 3 = slightly agree), nearly half (48.1%) of the students slightly, mostly, or strongly disagreed. Concerning the teaching of decision-making skills, most students thought that this subject was not sufficiently taught in medical school (IQR = 3–4, median 4 = slightly disagree). Finally, most students were afraid to encounter ethical and moral dilemmas in their future professional life, as 66.0% responded that they at least slightly agree (IQR = 2–4, median 3 = slightly agree). The responses correlate positively with the answers to the question “I think that physicians are often confronted with ethical and moral dilemmas in their professional life” (Spearman’s Rho = 0.19, *p* = 0.002), and there is an inverse correlation to the answers to the questions “I think that decision-making skills are sufficiently taught in medical school” (Spearman’s Rho = −0.13, *p* = 0.036) and “I feel sufficiently prepared to make a decision in the event of an ethical or moral dilemma” (Spearman’s Rho = −0.35, *p* < 0.001).

### Pandemic

Table 2 presents the results of the questions concerning pandemics. Most students felt prepared to make ethical and moral decisions during a pandemic (IQR = 2–4, median 3 = slightly agree) and were not afraid to deal with ethical and moral dilemmas in these times (IQR = 2–5, median 4 = slightly disagree); however, the participants stated that they were often worried about ethical and moral issues during the COVID-19 pandemic (IQR = 2–3, median 2 = mostly agree). Finally, students wanted more intense teaching on the ethical and moral issues during a pandemic (IQR = 1–3, median 2 = slightly agree).

**Table 2:** Summary statistics of questions related to pandemics

	1 Strongly agree	2 Mostly agree	3 Slightly agree	4 Slightly disagree	5 Mostly disagree	6 Strongly disagree	Missing	Overall score/mean	Median
I feel prepared to make ethical and moral decisions in times of a pandemic	6.0% (17)	25.1% (71)	*31.8%* *(90)*	23.3% (66)	9.2% (26)	3.9% (11)	0.7% (2)	3.16	3
I am afraid to deal with ethical and moral dilemmas in my professional life as a physician in times of a pandemic	7.8% (22)	17.3% (49)	15.5% (44)	*29.3%* *(83)*	20.5% (58)	8.8% (25)	0.7% (2)	3.64	4
I often worry about ethical and moral issues during the COVID-19 pandemic	19.1% (54)	*33.2%* *(94)*	22.6% (64)	14.5% (41)	7.1% (20)	2.5% (7)	1.1% (3)	2.64	2
I want more teaching on the ethical and moral issues during a pandemic	26.1% (74)	*31.8%* *(90)*	19.4% (55)	13.1% (37)	5.3% (15)	3.2% (9)	1.1% (3)	2.49	2

*COVID-19* Coronavirus disease 2019

### End of life

Table 3 gives an overview of the questions regarding the end of life. On the one hand, most of the participants felt prepared to deal with end of life dilemmas of patients as a physician (IQR = 2–4, median 3 = slightly agree) and were not afraid of having to deal with these dilemmas in their professional life (IQR = 2–5, median 4 = slightly disagree). On the other hand, the students did not think that they have sufficient legal knowledge to deal with end of life issues in their professional life (IQR = 3–5, median 4 = slightly disagree) and would have liked to have broader teaching on the ethical and moral issues of the end of life (IQR = 1–3, median 2 = mostly agree).

**Table 3:** Summary statistics of questions related to the end of life

	1 Strongly agree	2 Mostly agree	3 Slightly Agree	4 Slightly disagree	5 Mostly disagree	6 Strongly disagree	Missing	Overall score/Mean	Median
I feel prepared to deal with end of life dilemmas of patients in my professional life as a physician	5.3% (15)	22.6% (64)	*33.6%* *(95)*	22.3% (63)	11.3% (32)	4.2% (12)	0.7% (2)	3.25	3
I have sufficient legal knowledge to deal with end of life issues in my professional life	3.2% (9)	13.1% (37)	21.2% (60)	*30.7%* *(87)*	21.6% (61)	9.5% (27)	0.7% (2)	3.84	4
I am afraid of having to deal with end of life issues in my professional life as a physician	7.4% (21)	20.1% (57)	16.3% (46)	*26.1%* *(74)*	19.1% (54)	9.9% (28)	1.1% (3)	3.60	4
I would like to have more teaching on the ethical and moral issues of the end of life	30.7% (87)	*38.9%* *(110)*	19.8% (56)	5.3% (15)	3.2% (9)	1.4% (4)	0.7% (2)	2.15	2

### Gender differences

We evaluated the gender-specific differences of the responses. Most participants identified themselves as female (59.1%, *n* = 133), 39.6% (*n* = 89) as male. Due to the small, nonrepresentative sample size of participants who identified themselves as non-binary (*n* = 3), these questionnaires were excluded from the analysis.

Moreover, the responses of the 59 participants (20.5%) who did not specify their gender could not be implemented in the analysis.

Table 4 presents each question’s gender differences with the mean and median of both genders. The questions with statistically significant differences are in italic text and were marked as follows: * *p* < 0.05, ** *p* < 0.01, *** *p* < 0.001.

**Table 4:** Gender differences of answers to questions to the assessment of ethics teaching

	Asymp. Sig. (2-sided)	Mean (f/m)	Median (f/m)
*I think that ethics and morals are sufficiently covered in medical school*	0.030*	*3.44/3,06*	*4/3*
*I think that legal aspects about ethical and moral issues are sufficiently covered in medical school*	0.017*	*3.97/3.53*	*4/4*
*I feel sufficiently prepared to make a decision in the event of an ethical or moral dilemma*	0.002**	*3.69/3.16*	*4/3*
I think that physicians and professors take enough time to discuss ethical and moral issues with students	0.156	3.76/3.50	4/4
*I am afraid to encounter ethical and moral dilemmas in my future professional life*	<0.001***	*2.61/3.37*	*2/3.5*
I think that physicians are often confronted with ethical and moral dilemmas in their professional life	0.287	1.43/1.60	1/1
*I have sufficient legal knowledge to handle ethical and moral dilemmas*	0.005**	*4.24/3.79*	*4/4*
*I think that decision-making skills are sufficiently taught in medical school*	0.016*	*3.70/3.35*	*4/3*
*I want more emphasis on ethics and morals in medical school*	<0.001***	*1.91/2.48*	*2/2*
*I feel prepared to make ethical and moral decisions in times of a pandemic*	<0.001***	*3.41/2.83*	*3/3*
*I am afraid to deal with ethical and moral dilemmas in my professional life as a physician in times of a pandemic*	<0.001***	*3.41/4.23*	*4/4*
*I often worry about ethical and moral issues during the COVID-19 pandemic*	0.010*	*2.46/2.98*	*2/3*
*I would like to have more teaching on the ethical and moral issues in times of a pandemic*	<0.001***	*2.22/3.05*	*2/3*
*I feel prepared to deal with dilemmas at the end of life of patients in my professional life as a physician*	<0.001**	*3.46/2.92*	*3/3*
I have sufficient legal knowledge to deal with end of life issues in my professional life	0.780	3.83/3.80	4/4
*I am afraid of dealing with end of life issues in my professional life as a physician*	0.010*	*3.45/3.95*	*4/4*
*I would like to have more teaching on the ethical and moral issues concerning the end of life*	0.002**	*1.94/2.47*	*2/2*

*Asymp. Sig.* Asymptotic Significance, *(f/m)* (female/male), *COVID-19* Coronavirus disease 2019

Female students felt that ethics and morals as well as the legal aspects, were not sufficiently covered in medical school even more than the male students. They also assessed their legal knowledge to be more limited than their male colleagues. Moreover, the female participants felt less prepared to decide in the event of an ethical or moral dilemma and were afraid to encounter ethical and moral dilemmas in their future professional lives. Furthermore, they believed even more strongly that decision-making skills were not sufficiently taught in medical school and had a greater wish for more emphasis on ethics and morals in the curriculum than their male fellow students. Not only could a difference be found in the assessment of ethics teaching, but also the responses to the questions concerning pandemics. Female students felt less prepared to make ethical and moral decisions during a pandemic and had a greater fear of dealing with ethical and moral dilemmas in their professional life as a physician in times of a pandemic. Additionally, they worried even more about ethical and moral issues during the COVID-19 pandemic and had a stronger wish for broader teaching of ethical and moral aspects in times of a pandemic. Finally, the female participants felt less prepared to deal with dilemmas regarding end of life in their professional life as a physician, were more fearful of having to deal with end of life issues in their professional life as a physician and would have liked to have even more intense teaching of the ethical and moral issues at the end of life than their male colleagues.

### Preferences for medical ethics curriculum

Finally, we asked for the students’ wishes for ethics and morals teaching in the medical curriculum.

Table 5 presents the responses of the students to this question. Most students would have liked more emphasis on these topics (IQR 2, median 2 = mostly agree). The answers to this question showed a positive correlation to the responses to “I am afraid to encounter ethical and moral dilemmas in my future professional life” (Spearman’s Rho = 0.30, *p* < 0.001).

**Table 5:** Statistics for the question “I would like more emphasis on ethics and morals in medical school”

	1 Strongly agree	2 Mostly agree	3 Slightly Agree	4 Slightly disagree	5 Mostly disagree	6 Strongly disagree	Missing	Overall score/mean	Median
I would like more emphasis on ethics and morals in medical school	30.7% (87)	*40.6%* *(115)*	18.0% (51)	5.7% (16)	2.5% (7)	1.4% (4)	1.1% (3)	2.12	2

Next, we asked the students in which context they wanted an increased emphasis on ethics and morals. For this question, a selection of several answers was possible. The majority of students would have liked more teaching within the framework of case reports (70.7%), followed by discussions (62.5%), seminars in study groups (59%), lectures (30.4%), optional subjects (22.3%) and only 1.4% answered with “none”.

In the end, the students were asked about the topics on which they would like a deeper focus on the ethical and moral aspects during medical school in an open question. The most frequent answers were end of life/palliative care (*n* = 45), legal aspects (*n* = 23), health system/distribution of resources/influence of the economy (*n* = 12), general background/history of medicine (*n* = 12), and psychiatry (*n* = 10).

## Discussion

In light of the results of our study, we conclude that it is essential to emphasize the importance of bioethics as an integral part of medical education.

Past studies have shown medical students’ positive attitudes towards bioethics [18–21]. Interestingly, the results of our study show that the students were afraid to encounter ethical and moral dilemmas. This fear might stem from the awareness that decisions have consequences for the decision maker as well as the patients and families involved. The fear of encountering ethical and moral dilemmas might be tied to the feeling that the law is inadequately covered, as legal decisions can have serious professional and personal consequences.

This fear can also be found at the patients’ end of life, where futile life-sustaining treatment prolongs the life of patients. One of the reasons for this futility is the hesitance and inability of some doctors to decide due to their fear of legal consequences [22, 23]. Law should be a compulsory component of the curriculum of every medical school, providing the future doctors with the essential skills to make decisions in difficult situations.

Medical law is undoubtedly linked to bioethics and morality and forms the framework for decision making [24]. Laws are norms of specific ethical values that define the consensus of individuals’ social behavior in a community. Comprehensive ethical and moral training might not be enough to remove the fear of encountering dilemmas from the students, as law and ethics may diverge. The difficulty of deciding if something is legal because it is right or right because it is legal goes back as far as the ancient philosopher Plato in the Euthyphro dilemma [25].

Female students felt more afraid of dealing with ethical and moral dilemmas in their future professional life than male students and expressed a more significant wish for a deeper teaching of ethics. Similar results describing a greater demand from female students for broader ethics teaching were found in a study by Lehrmann et al. among medical students at the University of Mexico [20]. Furthermore, a survey of AlMahmoud et al. revealed that women endorsed the aims of ethics teaching more strongly [18]. Past studies showed that women were less confident in their knowledge and skills than their male colleagues [11, 12, 26, 27]. These differences in self-assessment might be a reason for the heightened demand for more ethics teaching by women and is likely to be an unconscious wish by men. To compensate for the gender differences, more gender-sensitive teaching in general and female role models in medical schools might help.

The students’ wish for a greater emphasis on ethics and morality in medical education could be found throughout all subtopics of our study. Over 90% of the students did at least slightly agree that they would have liked a greater emphasis on the teaching of ethics and morality in medical school.

Ethics and morality can be implemented in the curriculum in different ways. The participants of our study desired more intense teaching, especially in the framework of case reports (70.7%), discussions (62.5%), and seminars in study groups (59%). Past surveys attained similar results. In a survey at the Medical College of Georgia in 2018, among the most popular educational components for graduate level medical ethics, ethics case-based discussions (80.7%) and ethics discussions in small peer groups (62.7%) can be found [19]. Furthermore, among the five most desired methods for teaching ethics in a survey conducted in 2009–2013 at the United Arab Emirates University, case conferences, clinical rounds, and discussion groups of peers led by a knowledgeable clinician were mentioned [18]. Generally, integrated ethics education in medical curricula should be student centered and include problem-based teaching [28].

This study has potential limitations. One possible limitation of this study is its potential lack of generalizability, as the survey was conducted at one single medical school in Austria. Furthermore, the high percentage (20.5%) of missing data for the comparison of responses by gender might bias the results. Missing values are often unavoidable in research. Data missing not at random might result in a biased estimate of effect [29]. Finally, the data reflect the students’ opinions and self-assessment but cannot provide an objective assessment of their knowledge of ethics, morality, and medical law.

## Conclusion

Medical students will encounter various ethical, moral, and legal problems in their future professional lives. The COVID-19 pandemic and the ongoing discussions and legal changes concerning the end of life pose extreme challenges to healthcare workers. Solid training in ethics, morals, and medical law is indispensable to deal with these issues.

The Universal Declaration of Human Rights of the United Nations, which states an ideal of ethical standards, demands that “every organ of the society shall strive by teaching and educating to promote respect for these rights and freedoms” [30]. Furthermore, the World Medical Association demanded in its 51st World Medical Assembly in Tel Aviv in 1999 that “the teaching of medical ethics should become obligatory and be examined as part of the medical curriculum within every medical school” [31]. In response, in 2004, UNESCO established the Ethics Education Programme to support and foster ethics education in member states. Consequently, at the Medical University of Vienna, a Chair of Bioethics has been established as an institution responsible for bioethics education, ethical issues research and gender equality [32].

Implementing the students’ wishes in the curriculum pays off [33]. Ethics education should be customized to the students and the challenges they will encounter in their future professional life [34].

Basic knowledge of ethical principles is not sufficient for professional challenges. Extensive ethical and legal training during and after medical school is crucial to preserve patient rights, improve patient care quality and maintain healthcare workers’ mental health.
